# GigaDB: announcing the GigaScience database

**DOI:** 10.1186/2047-217X-1-11

**Published:** 2012-07-12

**Authors:** Tam P Sneddon, Peter Li, Scott C Edmunds

**Affiliations:** 1 GigaScience, BGI-Hong Kong Co. Ltd, 16 Dai Fu Street, Tai Po Industrial Estate, NT, Hong Kong

## Abstract

With the launch of *GigaScience* journal, here we provide insight into the accompanying database *Giga*DB, which allows the integration of manuscript publication with supporting data and tools. Reinforcing and upholding *GigaScience*’s goals to promote open-data and reproducibility of research, *Giga*DB also aims to provide a home, when a suitable public repository does not exist, for the supporting data or tools featured in the journal and beyond.

## Background

Internet pioneer Sir Tim Berners-Lee has stated: "Data is a precious thing and will last longer than the systems themselves” [1], and despite the challenges created due to data production in areas such as genomics growing at rates potentially faster than the ability to store and process it, attempts must still be made to capture and safeguard as much of these precious resources as possible. With the goals of *GigaScience* journal to maximize data reuse, dissemination, and transparency, having somewhere to host and curate all of the supporting data and tools surrounding this research is essential, and the *GigaScience* database, *Giga*DB (http://gigadb.org) is key to achieving this.

## Main text

As can be seen in *GigaScience’s* first issue, a research article on an epigenomics pipeline [2], in addition to having the raw data available in NCBI [SRP005934], also has this and all the supporting data (totaling 84 GB), such as the epigenomics tracks and the tools created for the pipeline [3], hosted in *Giga*DB. This dataset is linked and cited in the paper through a citable DOI (Digital Object Identifier), providing stability, and most importantly, additional discoverability and traceability through its ability to be tracked in the same manner as standard journal citations. Working and partnering with the British Library and DataCite consortium (http://datacite.org), these datasets are searchable and harvestable through their central metadata repository. Outside of the environmental sciences, data citation is still quite a new area, and we have worked closely with our publisher BioMed Central to ensure that citation of data follows DCC and DataCite best practice guidelines. In promoting the open-data movement, data is also released under the most open CC0 waiver, cutting any legal red tape [4], and maximizing its potential re-use. As *Giga*DB uses BGI’s extensive computing infrastructure, it has also been populated with datasets produced by BGI, much of it released in a citable form pre-publication.

Releasing data in this novel manner has had a number of successes to date, particularly spurring the crowdsourcing of data from the deadly 2011 *E. coli* 0104:H4 outbreak (also discussed in Mike Schatz’s commentary in this launch issue [5]) resulting in what has been termed “open-source genomics” [6]. For more on the background and mechanisms surrounding data citation, please see our recent correspondence [7] in the *BMC Research Notes* Data Sharing, Standardization and Publication series, using the release of the sorghum genome by *Giga*DB and publication in *Genome Biology* last year [8].

*Giga*DB currently comprises over 30 datasets. The largest of these is a hepatocellular carcinoma dataset [9], which consists of 15 Tb of normal and tumor raw data from 88 individuals. Additional data derived and processed from these same individuals, e.g. transcriptome sequence, can also be added to a DOI rapidly after their generation so users can immediately access the data from this ongoing project in a single, permanent place.

The goal of centralizing data and making it reproducible is exemplified by the mouse methylome dataset [3] in which we provide all data necessary to replicate the published results. This includes the raw fastq reads, bam alignment files, the Medusa software package, and the bigwig read-depth files. This and the sorghum study are excellent examples for future data submitters in regards to what can be done to not only comply with but also go beyond minimal journal data policies. Authors not only adhered to our standard journal editorial policies for genomics studies, with raw data deposition in one of the three INSDC databases and assemblies in Genbank, but the sorghum study also deposited additionally processed data to the dbSNP and dbVar databases. The methylome *Giga*DB page also includes data and associated files that do not have equivalent established repositories. The complementary system of releasing data through *Giga*DB and established repositories also has the advantage of making the data available much sooner than the staggered build releases of many of these databases, which can take several months.

The *Giga*DB website is continuing to evolve and the next version will be released later this year. Features in this version will include an extensive search interface allowing users to choose datasets and/or files for download/export by dataset type, file format, sample, species, DOI, external accession etc.

Although most published *Giga*DB datasets are genomic, we can accept any large-scale data including proteomic, environmental, and imaging data. Taking such a broad range of data types makes data interoperability an issue, and we have been working with the ISA-Commons community to see if *Giga*DB can capture study and assay metadata along with relationships between dataset components and take submissions using their ISA-Tab format [10]. We have a nice example in our first issue, with much of the data supporting the epigenomics pipeline paper stored in a more interoperable ISA-compliant manner [3]. Upcoming datasets will include gut metagenomic data and a *Drosophila* genomics workflow dataset. We would like to be as comprehensive as possible, especially in providing a home for data that is not represented in any of the major public databases/repositories, so we encourage you contact us if you have a dataset or tools you would like to submit to *Giga*DB.

Maximising the reuse of published data does not only involve its deposition, along with its metadata, into an open access repository in a standardised format. Results published in scientific articles also have to be reproducible so, for example, comparisons can be made with analyses on new research data [11].

In future editions of *GigaScience*, we will be working with authors to make the computational tools and data processing pipelines described in their papers available and, where possible, executable on an informatics platform. We hope that by making both the data and processes involved in their analysis freely accessible, this novel form of publication will help articles published in our journal to have a much higher impact in the scientific literature, and maximize their reuse within the community.

## Competing interests

All authors are employees of *GigaScience* and BGI.

## Author’ contributions

All authors have been working on *Giga*DB and have contributed to this editorial. All authors read and approved the final manuscript.
